# The role of aspirin in preventing gastrointestinal cancers

**DOI:** 10.1080/29937817.2024.2411460

**Published:** 2024-10-30

**Authors:** Maryam Ibrahim, Janusz Jankowski

**Affiliations:** a GI Department, University Hospitals Leicester, Leicester, UK; b Institute for Clinical Trials and Methodology, University College London, London, UK; c Tavistock and Portman NHS Foundation Trust, London, UK; d Dubai Medical College for Girls, Dubai, UAE

**Keywords:** cancer, chemoprevention, colorectal cancer, cancer prevention, esophageal cancer

## Abstract

Cancer remains an increasing global health issue and is projected to cause 50% of all global deaths by 2050. Gastrointestinal (GI) tract cancers currently account for approximately 25–30% of all cancer deaths. Consequently, there has been an emerging focus to optimize preventative measures for these cancers through varied avenues such as dietary change, public policy, screening/surveillance via endoscopy and most recently, chemoprevention. There is compelling evidence from both randomized controlled trials and population studies that the use of aspirin results in significant reduction of cancer occurrence in the colon (∼ 10–45% reduction). This could also be applicable to other GI sites, including the esophagus. The implementation of aspirin as a GI cancer prevention measure firstly requires; 1) the optimization of the risk/benefits involving regular uptake of aspirin and 2) the selection of an appropriate population that would benefit from aspirin use, either as a primary prevention or an adjuvant intervention for GI cancer management (and potentially other digestive tract cancers). National guidelines regarding aspirin use for GI chemoprevention are gradually being introduced as more evidence develops.

**Plagiarism and AI statement:** There were no sources copied and AI was not used in generating the content of this paper. Appropriate citation was also utilized.

## 1. Background

Gastrointestinal tract (GI) cancers, comprising oral, gastric, and colorectal malignancies, impose a huge burden on global health. These cancers are responsible for one-quarter of oncological diagnoses and approximately one-third of cancer-related deaths.^1^ Screening and surveillance pathways have helped reduce the incidence rate of certain forms of these cancers. For example, the use of colonoscopy to remove precancerous polyps has become the paradigm of an effective cancer-reducing intervention.^2^ However, interventional endoscopy isn’t a ‘magic bullet’ as it does not necessarily prevent cancer development in certain GI organs such as the stomach.^3^ In addition, issues such as poor patient adherence to endoscopy screening (∼ only 75%), financial cost (∼ $250-$1000 per procedure), and accessibility to adequate infrastructures in remote and deprived regions, hinder the optimization of screening programes. As a consequence of the aforementioned factors, only about 10% of GI cancers are diagnosed via screening programes. Therefore, evaluation of accessible and economical cancer prevention strategies remains the mainstay of current clinical aspiration.

Environmental factors such as a low-quality diet (i.e., dietary habit comprising of high saturated fats/trans-fats, highly processed, low fiber, high salt, and/or high sugar content meals) and smoking significantly influence the development of GI cancers.^4^ These risk factors bring about a chronic inflammatory state that creates the conducive microenvironment which enables the multi-step process preceding the development of malignancy in mucosal tissues. Consequently, the use of anti-inflammatory agents has garnered attention for a potential role in cancer prevention, particularly chemoprevention.

Chemoprevention entails the use of chemical agents (natural or synthetic) to inhibit or delay cancer development. Jankowski and Hawk summarized all key chemoprevention agents in their review, ranging from aspirin, acid suppression, hormone replacement therapy (HRT) to fish oil.^5^ These agents have the potential to impair dysfunctional protein activities that lead to initiation, promotion, and progression of tumorigenesis. As chemoprophylactic agents reduce malignant properties and tendencies, they could ultimately play a significant role in anticancer and systemic cancer treatment strategies.^6^

**Figure 1.**
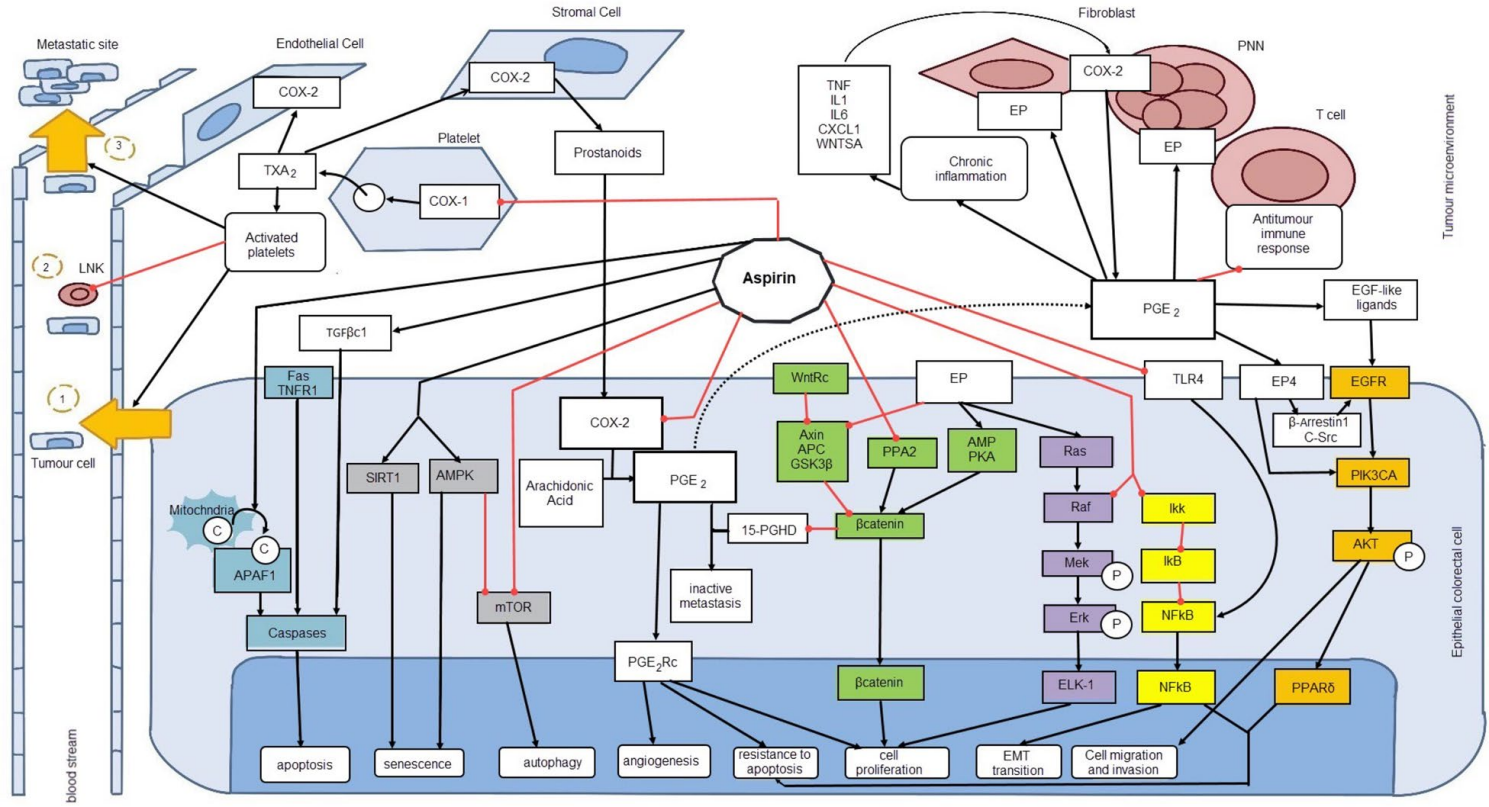
Effect of aspirin on cancer signaling pathways as explained by Grancher et al.^7^ Inhibitory effect of Aspirin on colorectal tumour cells. As illustrated by Grancher and colleagues, Aspirin inhibits intra- and extra cellular pathways that lead to tumorigenesis and metastasis. The Inhibition of PGE pathway, protein signaling pathways such as WNT which plays a key role in adenomatous polyposis coli (APC) are direct mechanisms that can hinder development of colorectal cancer. Furthermore, this image illustrates how aspirin plays key roles in the 8 hallmarks of cancer: apoptosis, evasion of apoptosis, senescence, autophagy, sustained angiogenesis, insensitivity to growth signals, cell migration and invasion. (8)

Importantly, aspirin and other non-steroidal anti-inflammatory drugs (NSAIDs) have chemoprotective properties against cancer via multiple pathways. Grancher and colleagues have illustrated these pathways (see Figure 1); most importantly, they demonstrated the ability of aspirin to irreversibly inhibit cyclooxygenase-2 (COX2) enzyme and the subsequent production of prostaglandin E2 (PGE_2_). COX-2 and PGE_2_ are inflammatory mediators released by tumour cells, and they induce apoptosis, cell migration, invasion, and angiogenesis, thus the progression of carcinogenesis.^7^

The aim of this paper is to discuss existing data that promotes the use of aspirin as a prophylaxis for GI cancers.

## 2. Method

English-language studies dating back to year 2006 were identified manually by the lead author. Publication search engines used in this search were Cochrane and PubMed. Key terms included in the search were gastrointestinal, esophageal, gastric, colorectal, and hepatobiliary cancer, and aspirin chemoprevention. A manual search of articles within the reference lists of publications was also conducted. This paper explores data which covers the use of aspirin as a chemoprophylaxis for GI and digestive tract cancers, the data reviewed are from randomized control trials (5), meta-analysis (3), cohort studies (2), and population-based studies (1), and the studies excluded are i) research that assesses non-aspirin anti-inflammatory agents as a chemoprophylaxis and ii) research that did not stratify the types of GI or digestive tract cancers. Existing guidelines on aspirin use as a chemoprophylaxis were identified via manual search.

## 3. Supporting evidence

Many studies have demonstrated that the use of NSAIDs lowers the risk of GI cancers (Table 1).^8^

**Table 1.** Relative risk of cancer reduction in varied digestive tract cancers with aspirin use (adapted from Bossetti et al.^9^).

Site of cancer	Overall relative risk	95% confidence interval	*P*-value
Colorectal	0.73	0.69 – 0.78	<0.001
Esophageal (Squamous cell)	0.67	0.57 – 0.79	0.006
Esophageal / Gastric cardia (Adenocarcinoma)	0.61	0.49 – 0.77	<0.001
Stomach	0.64	0.51 – 0.82	<0.001
Hepato biliary	0.62	0.44 – 0.86	<0.001
Pancreas	0.78	0.68 – 0.89	<0.001

Relative risks of regular aspirin use compared to non-aspirin use, in varied gastrointestinal cancer (GI) sites. As revealed in Bosetti and colleagues systemic review and meta-analysis of observation studies, aspirin use is associated with risk reduction across multiple GI cancer sites. They also found these values were consistent across gender and geographical regions (9).

### 3.1. Colorectal cancer (CRC)

Ren *et al.’s* UK Biobank cohort study with over 300,000 participants illustrated that regular use of COX-PGE_2_ inhibiting NSAIDS lowers the incidence of CRC by 36%.^9^ Aspirin has been the preferred NSAID due to its safer toxicity profile, and the vast knowledge base it has compared to other agents like celecoxib. A dose of aspirin as low as 81 mg daily inhibits COX-PGE_2_ mechanism by 45%, whereas other agents such as ibuprofen required much higher doses, such as 800 mg four times daily for a 63% decrease in PGE_2_ synthesis.^10^

Interestingly, regular aspirin use has been associated with protective changes in the gut microbiota. Particularly, aspirin intake prevents the growth of some pro-inflammatory bacteria that promote neoplastic transformation in the gut. Prizment *et al.’s* randomized control trial (RCT) supports this; they found a difference in the diversity and proportionality of varied bacteria within the gut microbiota of patients taking aspirin compared to the placebo cohort. For example, a larger proportion of *Akkermansia* bacteria was seen in patients taking aspirin. *Akkermansia* is known for its beneficial impact in colonic health.^11^

Rothwell and colleagues also reported that the prophylactic effects associated with aspirin were stronger with tumors within the proximal colon, the burdensome site where tumors are usually missed during colonoscopy.^12^ Similarly, Hull *et al.’s* ‘seAFOod Polyp Prevention Trial’ which evaluated the effect of omega-3 and aspirin on the reduction of colorectal adenomas, found that aspirin was the only chemoprotective agent that decreased the number of adenomas in the proximal colon. Colorectal adenoma was explored, given that it’s a well understood pathological predictor for CRC development, but they were unable to conclude that both drugs significantly reduced risk of CRC development in high-risk individuals. Nevertheless, both aspirin and omega-3 decreased the number of colorectal adenomas in participants at year 1 of colonoscopy surveillance.^13^ It is probable that significant benefit was not deduced as the research was run for only a short time (1 y). It is therefore likely that the study was underpowered as the benefits of chemopreventative measures are typically delayed by at least several years.

### 3.2. Esophageal and gastric cancers

In addition to CRC, other GI cancers have had favorable associations with low dose aspirin (LDA) therapy, Garcia *et al* showed that the use of LDA as low as 75 mg daily for at least 1 y was associated with a 41% and 54% risk reduction for esophageal and gastric cancer, respectively. Even though they found an increasing benefit with the duration of LDA use, there was no significant difference when comparing the duration of 1 to 3, 3 to 5 or >5 yrs.^14^ On the contrary, Rothwell’s meta-analysis showed that the benefit was strongest after 10 to 20 yrs follow up.^12^ This suggests the benefits of LDA use are much delayed than expected and it would ideally benefit individuals who are able to take aspirin for a lengthened duration of time. Thus, more research is required to help form an informative guideline which incorporates factors such as patient age and duration of aspirin use.

The ‘AspECT’ trial by Jankowski and colleagues evaluated the benefits of aspirin and proton pump inhibitors (PPIs) in precancerous metaplasia i.e., Barrett’s esophageal adenocarcinoma, and the mortality caused by both diseases. They found both drugs protected against all-cause mortality. However, high-dose PPI was the most protective of the two; it significantly prevented the progression of Barrett’s’ to cancer. The most impactful finding from this study was the additive chemoprotective effect of both drugs; the greatest protection and survival was found with the concurrent use of high-dose PPIs and LDA.^15^ Furthermore, serious adverse events from concurrent use of both agents were exceptionally low, less than 1%.

### 3.3. Hepatobiliary (HPB) and pancreatic cancers

There is also some evidence that aspirin might be beneficial in other digestive tract cancers. However, research here is not as extensive as that of GI luminal gut cancers. The meta-analysis conducted by Bosetti *et al* acknowledged that aspirin’s chemoprotective effect is present in HPB and pancreatic cancers. They found a stronger relative risk (RR) in liver cancers e.g., hepatocellular (HCC) than cholangiocarcinoma (Liver; RR of 0.71, 95% confidence interval (CI) 0.46 – 1.09, Cholangiocarcinoma; RR of 0.53 95% CI 0.24 – 1.14). Regarding pancreatic cancers an inverse relationship with aspirin was seen; a 20% deduction in pancreatic cancer risk was reported, which contradicts their previous study that found no significant reduction.^8^ In similarity to benefits seen in GI cancers, the protective effects seem to also increase with longer duration of aspirin use.^8^ Although liver cancers have the highest impact from aspirin (amongst HPB subgroup), the reports of chemo-preventative benefit are inconsistent and has been identified mainly in hepatitis B-virus related HCC.^16^

Controversial findings seen in HPB, and pancreatic cancers could be due to the more diverse etiological factors that progress into malignancy. In correspondence to GI cancers, risk factors such as advancing age, alcohol and smoking predispose the chronic inflammatory changes that lead to neoplastic transformation. However, HPB and pancreatic cancers have a wider range of risk factors such as diabetes mellitus, cholelithiasis, and infections such as hepatitis and liver fluke.^17^ These all potentially have varied mechanisms which may or may not incorporate COX-2 pathway. Therefore, more research is still required to review the potential of chemoprophylaxis in this subgroup. Other chemical agents, that have been associated with reduced risk of these cancers include statins and metformin.^18^^,^^19^

## 4. Potential limitations for regular aspirin use

So far, the main hesitancy towards the consideration of aspirin as a prophylaxis for GI cancers is the risk of hemorrhage. Ingestion of aspirin leads to the inhibition of thromboxane-A2 platelet aggregation; as a result, bleeding time is prolonged, and this could last hours to days as this inhibitory process could persist throughout the platelet cells’ life span.^20^ Major hemorrhagic events come with significant complications such as bleeds which require transfusions, intracranial hemorrhage that can lead to neurological deficit or death. Thus, the benefit of regular LDA use is becoming increasingly questioned in day-to-day clinical practice, especially its use as a primary prevention for cardiovascular diseases (CVD) or strokes in the older population.

Aspirin and other NSAIDS also cause GI wall damage, leading to ulcers and predisposition to GI bleeds. In fact, it has been advised that routine prescription of doses higher than 81 mg should be avoided due to dose-dependent adverse effects.^21^ However, in the aforementioned AspECT Trial, the combined use of LDA and PPIs had an exceptionally low GI bleed rate ∼ <0.1% over 9 yrs.^15^

## 5. Existing guidelines and promising studies

The use of aspirin in GI cancer prevention has been incorporated in some countries, albeit with some variations in recommended dosage and target populations. Australia implemented LDA use for CRC prevention in their general population in 2017. There, Cancer Council Australia released a guideline recommending daily use of 100 to 300 mg aspirin for 2.5 to 5 yrs amongst people aged 50 to 70 yrs.^22^ Thereafter, RCTs such as the ‘Should I take Aspirin trial’ (SITA) trial were initiated to guide patients’ decision on aspirin use. Although, the SITA trial revealed that exposure to the decision aid they designed prompted participants to discuss aspirin with their care provider, there was no statistical evidence to show that it led to an increase in the uptake of regular aspirin.^23^ This suggests that clinical knowledge is not enough to guide patients’ informed choices, and these clinical encounters could benefit from the addition of a clinical scoring system. Whereby, clinicopathological risk factors (such as nutritional status or predisposition to chronic GI inflammation) that estimate patients’ likelihood of developing colorectal cancer are used alongside decision aids.

In England, the National institute for health and Care Excellence (NICE) released a new guideline for an off-label use of daily aspirin for people living with lynch syndrome to decrease their risk of CRC. However, their recommended dose has not been decided and current practice is to use 150 to 300 mg aspirin.^24^ The United States proposes aspirin prophylaxis in a multi-systemic fashion. They have initiated a dual recommendation where individuals aged 50 to 59 yrs with a ≥10% likelihood of developing CVD in 10 yrs, and who also have an inherited risk of colorectal cancer are advised to take up LDA for at least 10 yrs for primary prevention of CVD and CRC.^25^

Within the world of oncology, the impressive benefits of aspirin have also been noted in non-GI cancers such as breast and prostate cancer, as its use have led to better cancer outcomes.^26–28^ Coyle *et al* are currently running a phase III trial, the ‘ADD-aspirin trial. This trial is assessing the use of 100 and 300 mg aspirin for prevention of cancer recurrence. Patients treated for early-stage breast, colorectal, gastric, esophageal, and prostate cancers have been recruited and will be revaluated after ≥5yrs of aspirin use.^29^ Such an integrative trial is promising, as it can help weigh risk and benefits, and thus help with a much distinct guideline, as people who have previously had cancer have a substantial risk of relapse or developing new cancers.

The 5-year survival rate for localized and distant CRC is 91% and 13% respectively.^30^ The sizeable difference in the estimated outcome between these CRC extremes illustrates the significant burden posed by disease progression. On this account, it would be worthwhile to generate further guidelines where prognostics factors such as the manner of surgery (radical or non-radical), genetic mutations such as TP53, and/or basic blood tests such as albumin level are incorporated to recommend aspirin prophylaxis to improve disease outcome. Several scoring systems such as Gustave Roussy Immune (GRIm-Score), MD Anderson Cancer Centre (MDACC), Royal Marsden Hospital (RMH) scores that use diverse clinicopathological parameters have been generated and are currently being used to guide prognostication and patient selection for clinical trials.^31^^,^^32^

## 6. Discussion

Aspirin is readily available and is one of the most used drugs globally. Its potential as a preventative measure against GI cancers is supported by a growing body of evidence. Aspirin’s beneficial association is not limited to chemoprevention; its preventative effects are well known in ischaemic diseases such as stroke and myocardial infarction.^33^ Thus, regular aspirin use benefits overall morbidity and mortality, and possibly lengthens an individual’s lifespan.

The main limitation with the use of aspirin is its link with hemorrhage. However, there are many factors to bear in mind when understanding the bleeding risks. Some of these risks, such as GI hemorrhage are curbed with the use of PPIs; their synergistic uptake has been proven to enhance chemoprevention in cancers such as esophageal cancer. Even though aspirin inhibits coagulation cascades, its use is usually not the primary cause of bleed, and research has shown that other factors, such as *Helicobacter pylori* exposure and chronic hypertension, exacerbate the clinical impact of hemorrhaging arterioles and capillaries.^34^

So, we know that aspirin works; we can appreciate that a dose as low as 75 mg daily is effective; and furthermore, we know that the longer a patient uses aspirin, the more likely they will gain the chemoprotective benefits over time. As seen in current studies, a 10-year duration is associated with the reduction of GI cancer risk; thus, the ideal population would be middle-aged patients who have been deemed to have low bleeding risk following a risk assessment by their care provider. Regarding recommendations for HPB and pancreatic cancers, there are some benefits, but more research is still required to deduce considerable conclusions.

On a molecular level aspirin impacts on multiple cancer signaling pathways and studies have also illustrated the benefits of aspirin across many digestive tract cancers. Although for some cancers the benefits are more modest than others. The existing aspirin-GI chemoprevention guidelines discussed earlier have different recommendations for aspirin use. For example, in Australia it is recommended for a specific age bracket, whilst in the UK it is recommended for patients with lynch syndrome. This further emphasizes the need for more research on how to best implement aspirin chemoprophylaxis. All aspects considered it remains clear that aspirin use makes sense. As deduced from the SITA study more measures are also required to reinforce patient decision aids and thus, successfully implement aspirin introduction for GI cancer prevention. Additionally, benefits of aspirin in established cancer patients are also being uncovered.

Whilst the facts discussed in this paper are from published sources, there are some limitations that should be acknowledged. Firstly, no statistical analysis was performed and the discussions in this paper are based on previously published research. Secondly, a few of the reports were from case control studies which are prone to biases such as recall, selection, or interviewer bias. Also, similar to any research that looks at the relationship between factors such as lifestyle or medication history to a specific disease, there is usually a risk of healthy population bias where participants are possibly healthier than the general population. Therefore, the presence of other external factors could potentially augment the good results seen from regular aspirin use.

In conclusion, the use of LDA as a chemoprophylaxis has a huge role in the future of gastrointestinal cancer prevention schemes. Low-cost cancer prevention schemes are readily used in other medical fields e.g., the use of retinoids and sunscreen in dermatology, for skin cancer incidence.^35^^,^^36^ Therefore, application of these chemoprophylaxis findings into day-to-day clinical practice is promising, especially for patients who are at increased risk or who have previously had GI cancers. There are still some loopholes in the interpretation of the existing studies. Firstly, an agreed dose needs to be determined; more work is still required in the future to help determine the lowest effective dose that would inhibit micro-oncogenic inflammatory processes without causing harm. Secondly, the complexity of drug history and existing comorbidities in middle aged adults’ results in some level of uncertainty regarding the exact effect of aspirin. For instance, patients who are on regular aspirin, tend to be on statins also, which both have an influence in GI chemoprevention. Thus, these confounding factors need to be considered in future research.

## 7. Take home message

Avoid aspirin if you have had an allergy to it or have risks of bleeding in the GI tract (GI ulcers or Inflammatory bowel disease) or brain (aneurysm or high blood pressure).

Consider taking aspirin for 5–10 yrs, sometime between 50–70 yrs of age, to maximize the benefits and minimize the risks.

Combination of aspirin and acid suppression agents seems synergistic in esophageal cancer.

## Data Availability

Data sharing is not applicable to this review as the report was based on existing data, thus no new data were generated or analyzed.
